# Mode II Behavior of High-Strength Concrete under Monotonic, Cyclic and Fatigue Loading

**DOI:** 10.3390/ma14247675

**Published:** 2021-12-13

**Authors:** Henrik Becks, Martin Classen

**Affiliations:** Institute of Structural Concrete, RWTH Aachen University, 52074 Aachen, Germany; mclassen@imb.rwth-aachen.de

**Keywords:** fatigue, high-strength concrete, mode II loading, experimental investigation, shear test

## Abstract

An economically efficient yet safe design of concrete structures under high-cycle fatigue loading is a rather complex task. One of the main reasons is the insufficient understanding of the fatigue damage phenomenology of concrete. A promising hypothesis states that the evolution of fatigue damage in concrete at subcritical load levels is governed by a cumulative measure of shear sliding. To evaluate this hypothesis, an experimental program was developed which systematically investigates the fatigue behavior of high-strength concrete under mode II loading using newly adapted punch through shear tests (PTST). This paper presents the results of monotonic, cyclic, and fatigue shear tests and discusses the effect of shear-compression-interaction and load level with regard to displacement and damage evolution, fracture behavior, and fatigue life. Both, monotonic shear strength and fatigue life under mode II loading strongly depend on the concurrent confinement (compressive) stress in the ligament. However, it appears that the fatigue life is more sensitive to a variation of shear stress range than to a variation of compressive stress in the ligament.

## 1. Introduction

The characterization of concrete fatigue has been a research topic for several decades and has been extensively investigated and discussed in literature. A variety of fracture mechanics and phenomenological approaches are available to describe and predict the damage evolution and propagation of normal- and high-strength concrete [1,2,3,4,5,6,7]. However, the basic mechanisms underlying the fatigue behavior of quasi-brittle materials are still not sufficiently understood, and thus, important aspects like the influence of load sequence [8,9], load frequency and temperature [10], or moisture content [11] remain unacknowledged.

A promising hypothesis, originally postulated in [12] and further refined in [13], states that the evolution of fatigue damage at subcritical compressive load levels is governed by a cumulative measure of shear sliding. The model is based on experimental observations presented in [14] and incorporates inelastic mechanisms explicitly in its thermodynamic potentials so that it is able to reflect the triaxial stress redistribution within a material zone that takes place under oscillating subcritical loading. To validate this hypothesis experimentality, a new test setup was developed to characterize the behavior of concrete under defined shear loading and traceable compressive stress. A comprehensive test program consisting of 46 monotonic, cyclic, and fatigue shear tests was conducted for in-depth analysis.

The aim of this investigation is the evaluation of the fundamental material behavior of high-strength concrete under mode II fatigue loading and the determination of the influence of a concurrent compressive load. Particular attention will be paid to the development of displacement and damage, the fracture behavior, as well as the fatigue life.

## 2. A Brief Overview of Shear Testing

Shearing is the most common mode of failure in rock engineering [15]. Therefore, early investigations on the shear strength of this quasi-brittle material were carried out as early as the 1930s [16]. While these early studies most likely investigated the chemical bond forces rather than the mode II fracture behavior known from fracture mechanics [17], the significant influence of compressive forces perpendicular to the fracture surface (confinement) was already described at that time. The first systematic experimental studies on the fracture behavior of rock under mode II loading were described by Robertson in the 1950s [18]. Since then, the material behavior of rock under shear loading has been intensively researched, yielding various test setups (e.g., [15,19,20,21,22,23,24,25,26,27,28]).

**Figure 1:**
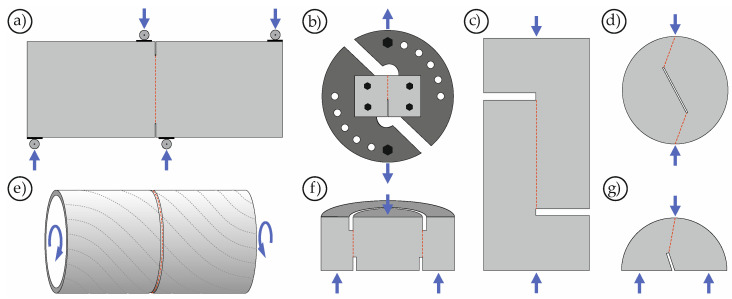
Different test setups for mode II testing: (**a**) Anti-symmetric four-point bending test (e.g., [30]), (**b**) Notched Arcan specimen (e.g., [28]), (**c**) Short beam/core in compression (e.g., [26,34]), (**d**) Cracked straight-through Brazilian disc (e.g., [35,36]), (**e**) Notched thin-walled tube (e.g., [33,37]), (**f**) Punch through shear test (e.g., [22,24,38,39]), (**g**) Notched semi-circular bend (e.g., [35,36]).

Already in 1906, Zipkes presented the first investigations on the shear strength of concrete [29]. First systematic studies on mode II fracture of concrete were published in the 1980s (e.g., [22,30,31,32,33]). Since concrete, similar to rock, is a quasi-brittle material, it was possible to draw on the extensive experience gained from investigations on rock. Figure 1 shows examples of different test setups that were used in the past to investigate the material behavior of concrete and rock under mode II loading.

Mode II fracture can be divided into two categories: pure mode II fracture under pure shear loading and compressive shear fracture under compressive shear loading [40,41]. The complexity in all experiments is to prevent mode I fracture and provoke cracking in pure sliding. According to Lin [40], the punch through shear test (PTST) is particularly well suited for inducing a mode II (compressive shear) fracture.

The PTST in its cylindrical form was originally developed by Luong [22,42,43,44]. Since then, it has been adopted and extended by many researchers. Backers, for example, studied the mode II fracture toughness of rock under simultaneous compressive loading [23,24,45,46], Montenegro investigated the shear strength of concrete under confined conditions [38,47,48] and Forquin & Lukic mainly investigated the material behavior under mode II impact loading [39,49]. In all investigations, it was found that the material behavior under mode II loading strongly depends on the concurrent compressive loading. The positive influence of normal stresses on shear strength has been acknowledged for a long time [50]. In his theoretical investigations Melin describes that a high normal compressive force, acting perpendicular to the direction of loading, promotes mode II cracking [51]. Since a compressive normal force is always applied to the shear surface in PTST (due to dilation of the concrete), this setup is ideal for mode II testing.

## 3. Experimental Program

### 3.1. Material Properties

Within the scope of our own investigations, a high-strength, self-compacting concrete with a maximum aggregate size of 4 mm was used. Modulus of elasticity and splitting tensile strength were determined on cylinders (*h*/*d* = 300 mm/150 mm) to 33,010 MPa and 3.4 MPa, respectively. Compressive strength was tested on cube specimens (*a* = 150 mm) to 101 MPa. All material properties were determined after 28 days of curing. The shear specimens were cast in several batches together with prismatic material samples (*l*/*h*/*d* = 160 mm/40 mm/40 mm). To prevent significant deviations in material properties, the flexural and compressive strength were determined using these prismatic samples on the day of testing.

### 3.2. Specimen Geometry and Preparation

**Figure 2:**
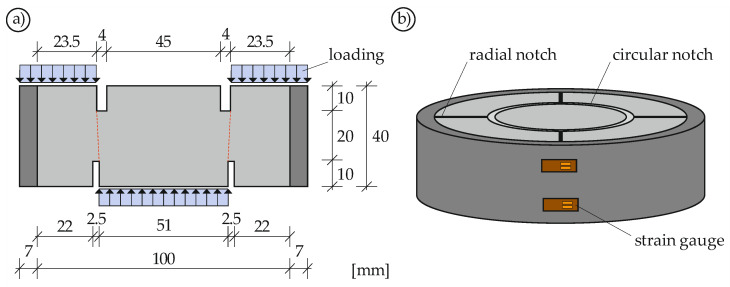
PTST Cross-section (**a**) and isometric view (**b**).

To investigate the monotonic and cyclic material behavior of concrete under mode II loading, the PTST, already presented in Section 2, was applied. Considering that fatigue loading should be as uniform and reliable as possible, the straightforward test setup is beneficial. The lower and upper circular notches were designed for the inner diameter of the lower notch to correspond to the outer diameter of the upper notch in order to obtain a straight cylindrical fracture surface (Figure 2). To avoid brittle material behavior due to radial mode I cracking, the concrete was cast in a steel ring. By measuring the strain of the steel ring, the confinement induced by the dilation of the concrete can be determined. The outer concrete ring was weakened with 4 to 8 radial notches to prevent self-confinement, which would bias the strain measurement.

Before concreting, the radial notches were prepared by applying non-adhesive polyethylene foam spacers. After concreting, the specimens were cured for one day before being stored under water for seven days. The circular notches were then cut with a core drill and the top surface of each specimen was ground to be parallel to the bottom surface.

### 3.3. Test Setup, Instrumentation and Test Procedure

**Figure 3:**
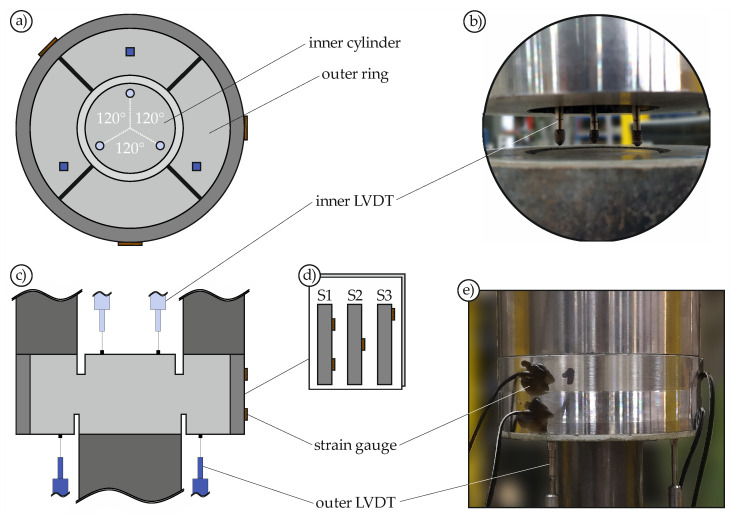
Test setup and instrumentation: top view (**a**), detail of inner LVDT (**b**), cross-sectional view (**c**), strain gauge variations (**d**), and side view (**e**).

Not only the confinement, but also the relative displacement between the inner cylinder and the outer ring has to be measured. For this purpose, the setup shown in Figure 3 was developed. To detect tilting of the inner cylinder, the displacement was measured using three linear-variable displacement transducers (LVDTs) each at the bottom and top. The upper LVDTs were placed inside the steel ring (Figure 3b). The strain gauges were placed at three different locations around the circumference of the steel ring, ensuring that one was always on the notch and two were between the notches. In addition, the strain gauges were attached in different ways (Figure 3d) to record the stress distribution along the specimen height.

### 3.4. Loading Scenarios

To systematically investigate the structural behavior under monotonic, cyclic, and fatigue mode II loading, a comprehensive test program, already established in [9], was applied (Table 1). The fundamental intention of such experimental design is to obtain precise information about the fracture behavior, the damage development, and the nonlinear shear-confinement interaction. Furthermore, it provides a sound basis for a systematic calibration and validation procedure of numerical models and engineering design rules. First numerical studies using the presented experimental results can be found in [52].

*LS1*: The first loading scenario introduces a monotonically increasing displacement at the rate of 0.1 mm/min (controlled by the displacement of the test piston) until a maximum displacement of 6 mm. This allows the maximum shear strength τ_max_ to not only be derived as a function of confinement σ_c_, but also the complete stress-displacement curve. In addition, fracture behavior, size effect, and the influence of the radial notches can be investigated.

*LS2*: In the second loading scenario, a displacement-controlled cyclic loading is applied with an increasing displacement. A total of five unloading steps with a constant rate of 0.2 mm/min (controlled by the displacement of the test piston) are performed. LS2 provides detailed information on the post-peak loading and unloading behavior of the stress-displacement curve. Besides energy dissipation, which can be evaluated using the hysteretic loops, it is also possible to determine the strength degradation within a loading cycle.

*LS*3: The third loading scenario comprises load-controlled cyclic loading in a maximum of 130 cycles with a frequency of 0.05 Hz. *S*_max_ starts at 0.50 · τ_max_ and is increased by 0.05 every 10 cycles until failure. *S*_min_ remains constant at 0.05. This loading scenario allows for fundamental investigation of the pre-peak loading and unloading behavior at subcritical load levels without having to perform thousands of cycles. Moreover, the influence of small load jumps and the damage accumulation mechanisms at subcritical load levels can be investigated.

*LS4*: In the last loading scenario, fatigue loading with constant amplitude is applied at a frequency of 5 Hz. Four different configurations are investigated. *S*_max_ varies between 0.85 and 0.75, while *S*_min_ ranges from 0.05 to 0.20. An overview of all load combinations is shown in Table 2. The goal of LS4 is to investigate concrete fatigue under mode II loading with constant amplitude in terms of fatigue life, displacement development, confinement-shear stress interaction, and load level. For this purpose, fatigue-displacement curves, Wöhler curves, and fatigue-confinement curves are evaluated.

## 4. Experimental Results and Discussion

### 4.1. Monotonic Behavior

#### 4.1.1. Calculation of the Confinement

When a body is subjected to shear, the volume increases, a process known as dilation. If this dilation is restrained, e.g., by an outer steel ring, a confinement emerges inside the specimen. In the specimen employed in this study, this confinement acts like a compressive stress perpendicular to the shear surface. Since various studies (e.g., [38,39,48,49,53,54]) have already shown a direct dependence between confinement and shear strength, it is essential to measure the relationship between these two stresses. In the present investigation, similar to [39,49,53,54], Barlow’s formula (Equation (1)) was used to calculate the confinement based on the steel ring strain ε_s,mean_.

(1) $$\sigma_{s}=\varepsilon_{s,\mathrm{mean}}\cdot E_{s}=\frac{\sigma_{c,\mathrm{ring}}\cdot \left(d+s\right)}{2\cdot s}$$

The modulus of elasticity of steel *E*_s_ is assumed to be 210,000 MPa. The thickness of the steel ring *s* and the diameter of the concrete cylinder *d* can be found in Figure 2. The confinement σ_c,ring_ describes the pressure acting on the inner steel ring surface *A*_s_. To obtain the true confinement σ_c_, σ_c,ring_ must be related to the shear surface *A*_Liga_ (Equation (2)).

(2) $$\sigma_{c}=\frac{\sigma_{c,\mathrm{ring}}\cdot A_{s}}{A_{\mathrm{Liga}}}$$

#### 4.1.2. Stress-Displacement Curve

**Figure 4:**
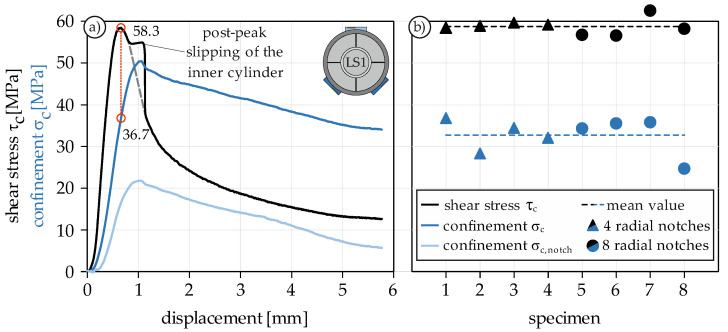
Stress-displacement curve under monotonic mode II loading (**a**) and maximum shear stress with corresponding confinement (**b**).

Using these basics, it is possible to determine the confinement as a function of the displacement. Figure 4a shows an example of an experimentally determined stress-displacement curve. A similar behavior can be observed for all conducted PTST: The shear stress increases steadily and steeply until the maximum shear strength is reached after a displacement of 0.5 to 0.6 mm. Then, the load declines continuously. After a load drop of 5 to 10%, the inner cylinder of the specimen slightly slips, which can be recognized by the sudden increase in displacement and the accompanying decrease in load. However, the dashed gray line in Figure 4a clearly indicates that the course of the stress-displacement curve is not affected by this slipping. Subsequently, the shear stress asymptotically approaches a limit value of roughly 10 to 20 MPa, which can be substantiated with friction between inner cylinder and outer ring.

#### 4.1.3. Interaction of Shear Strength and Confinement

Since the shear surface decreases with increasing displacement, the surface reduction must be considered when calculating the shear stress and confinement. To determine the confinement σ_c_, the two strain measurements between the radial notches (dark blue) were averaged (Figure 4). In direct comparison to the calculated confinement on the notch σ_c,notch_ (light blue), σ_c_ was in all tests about twice as large, which could be attributed to the bending of the steel ring. Given that the dilation increases even after the maximum shear strength is reached and only decreases when the inner cylinder slips, it is more rational to examine the confinement at the level of maximum shear stress. For specimen 1, the confinement is 36.7 MPa at a maximum shear strength of 58.3 MPa. Figure 4b shows the maximum shear strength and corresponding confining stresses of all conducted monotonic tests. The shear strength shows very little scatter and averages 58.7 MPa, whereas the corresponding confinement averages 32.7 MPa. The confinement appears to be equally high throughout all tests, even if no clear trend between confinement and maximum shear stress is evident. The number of radial notches has apparently no influence on the confinement, thus it can be assumed that four notches are sufficient to prevent self-confinement.

**Figure 5:**
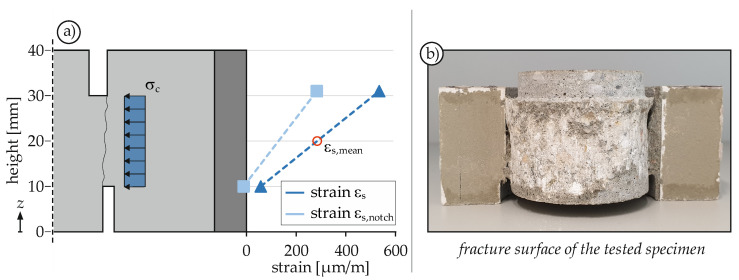
Strain distribution of a specimen with four radial notches at peak load (**a**) and fracture surface after testing (**b**).

All confining stresses in Figure 4 were calculated based on the strain measurements at mid specimen height. As already described in Section 3.3, the strain gauges were attached in three different ways. This made it possible to identify the strain distribution along the specimen height. Figure 5a shows the determined strain distribution. As can be clearly seen, the strain increases towards the support ring. This is due to the bending of the specimen, which has an obvious influence on the stress distribution despite the short supporting span. To be able to compare the determined confinements nevertheless, the strains were calculated to the mid specimen height (20 mm). It was assumed in a simplified way that this mean strain ε_s,mean_ acts constantly along the entire height (Figure 5a).

#### 4.1.4. Fracture Behavior

Figure 5b shows the fracture surface of a specimen with four notches due to monotonic loading. Clearly, the fracture surface is formed between the inside of the lower notch and the outside of the upper notch. Due to the chosen geometry, this results in an almost straight shear surface. Apart from this shear surface, no other visible cracks develop: neither in the outer concrete ring (“wing cracks” [49] or “doughnut fractures” [23,24,45]), nor in the inner concrete cylinder (“wing cracks” [23,24,45]), or additional cracks along the shear surface (“macroscopic en échelon fractures” [23,24,45]). The reason for this could be the significantly larger maximum aggregate size, which results in a different fracture behavior. This behavior is more comparable with the granite tests described in [23,24,45], which have a maximum grain size of 5 mm. 

#### 4.1.5. Size Effect

**Figure 6:**
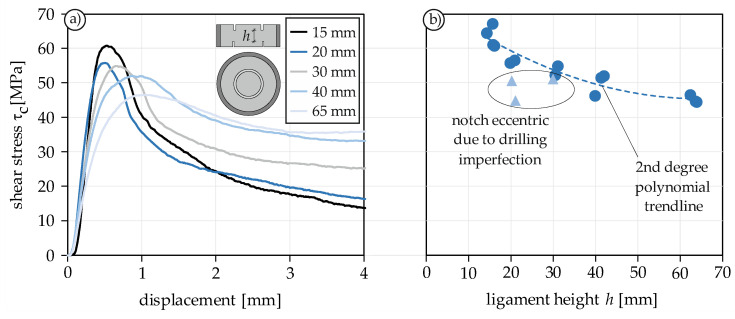
Stress-displacement curves of PTST with various Ligament heights under monotonic mode II loading (**a**) and maximum shear stress of all conducted tests (**b**).

To investigate the influence of the specimen height on the shear strength, 17 monotonic shear tests with five different ligament heights *h* (15–65 mm) were carried out. Since tests with small shear lengths are subject to significantly larger scatter due to the heterogeneity of the concrete, the minimum ligament height was chosen to be about four times the maximum aggregate size (15 mm). The maximum ligament height was adapted to the capacity of the testing machine and set to 65 mm. Since only the size effect was of interest and the confinement was not the objective of the study, the arrangement of radial notches was omitted. Figure 6a shows the stress-displacement curves of five shear tests with different ligament heights. The maximum shear stress decreases with increasing ligament height. This is known as size effect. In addition, the curve flattens, the peak is shifted to the rear and the residual stress due to friction increases. The post-peak slipping of the inner concrete cylinder, which is visible in Figure 4a, is absent in these tests due to the lack of notches. Figure 6b shows the shear strength of all conducted tests. The light-blue triangles represent tests which had considerable eccentricities of the upper and lower notches due to drilling imperfections. This underlines the importance of accurate specimen preparation, since even small imperfections can have a pronounced influence on the load transfer and final shear strength.

The present investigation shows a clear impact of the ligament height on the shear strength and stress-displacement curve, as already shown in [30,55]. According to [37], the reason for this are the stress peaks at the edges of the ligament, which are caused by discontinuities and falsify the determined average shear stress. All statements on material behavior under shear loading should therefore always be associated with the ligament height. Alternatively, a test setup should be used where a constant shear stress over the ligament height can be applied and is thus not affected by the size effect [33,37].

### 4.2. Post-Peak Cyclic Behavior

#### 4.2.1. Stress-Displacement Curve

**Figure 7:**
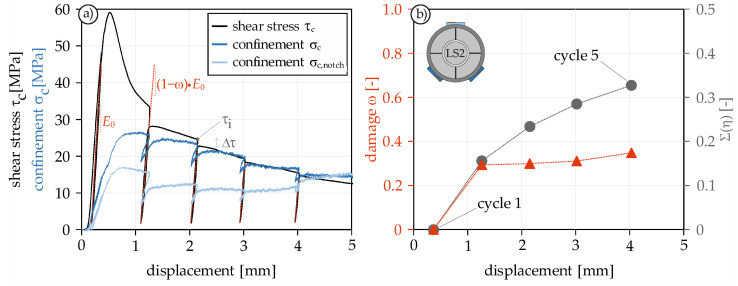
Stress-displacement curve under cyclic mode II loading (**a**) and the calculated degradation effects (**b**).

One of the most important aspects of investigating concrete under fatigue loading is to identify the dissipative mechanisms. Since this is a complicated and time-consuming task for high-cycle fatigue loading, low-cycle tests are suitable, where displacement-controlled loading is applied with unloading steps at different displacements. Figure 7a shows the result of such a test with five loading and unloading steps.

As in series LS1, the widening of the steel ring during cycling loading was measured and converted into the confinement. With each unloading, the confinement slightly drops. However, since the displacement does not return to zero at any of the post-peak unloadings, the dilation is also maintained. Thus, the confinement drop never exceeds 30% (Figure 7a). Noteworthy is that the unloading stiffness of the confinement usually is significantly higher than the loading stiffness.

#### 4.2.2. Evaluation of the Degradation Effects

During cyclic loading, various dissipative mechanisms occur which are associated with material degradation. They are an important indicator for the remaining lifetime of the specimen. During the evaluation of the cyclic tests, two degradation effects could be identified. One is the decrease in unloading stiffness. In previous research [9,56,57], this decrease is usually used to describe the damage development and is formulated by the damage parameter ω (Equation (3)).

(3) $$\omega =1-\frac{E_{i}}{E_{0}}$$

Figure 7a reveals that the stiffness initially increases slightly after pre-peak unloading and does not decrease. This behavior under cyclic loading of shear tests is already known from [46,58]. *E*_0_ therefore refers to the stiffness of the first unloading and not the initial stiffness. With the first post-peak cycle, the damage increases to about 0.3 and remains approximately constant thereafter (Figure 7b). The fact that there is no larger decrease in stiffness, unlike in cyclic compression or tensile tests, is due to the confinement, which compresses the shear surfaces even after the maximum shear strength is exceeded, thus preventing a severe decrease in stiffness. Similar results were also described in [10], where the stiffness did not decrease more than 75%. As an indicator of damage, the parameter ω is therefore not very convenient and a comparison based on the stiffness decrease would be much more suitable.

The second degradation effect is the reduction Δτ of the load at the beginning and end of a cycle. Based on this reduction, it can be seen that there are significant dissipative processes during cyclic loading despite the very small decrease in stiffness and hardly any hysteretic loops. For a better understanding of the magnitude and development of this damage, the reduction Δτ was related to the initial load of each cycle τ_i_ to obtain the parameter η (Equation (4)). Figure 7b shows the cumulative evolution of η. An under-proportional growth of the relative cumulative reduction can be seen, which leads to the conclusion that the shear stress degradation per cycle decreases with increasing displacement. The reason could be the increasing contribution of friction with larger displacement, which is determined exclusively by the confinement. Therefore, it is not related with the pure shear resistance of the specimen.

(4) $$\eta =\frac{\Delta \tau }{\tau_{i}}$$

### 4.3. Pre-Peak Cyclic Behavior

**Figure 8:**
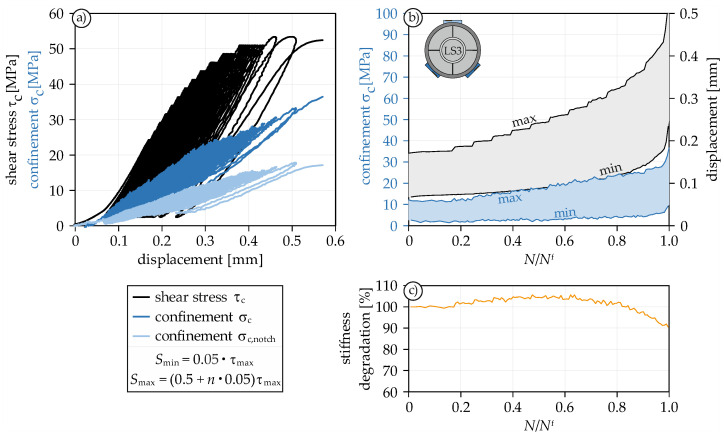
Stress-displacement curve under cyclic step-wise increased mode II loading (**a**), fatigue-displacement curve with corresponding confinement (**b**), and stiffness degradation (**c**).

After investigating the post-peak behavior under cyclic loading in the LS2 series, LS3 is designed to investigate the pre-peak behavior. In order to initiate a quicker failure, the upper load *S*_max_ was initially set to 0.50 and increased by 0.05 every 10 cycles. The stress-displacement curve of one LS3 test is shown in Figure 8a.

In addition to the clearly visible loading jumps, the nonlinear deformation behavior can also be seen in the stress-displacement curve (Figure 8a). The confinement rebuilds with each loading and decays again with each unloading. Thus, there is no constant confinement during cyclic loading, but an in-phase loading and unloading.

The development of the displacement as a function of fatigue life clearly shows the loading jumps as well (Figure 8b). As already known from compression and tensile tests under cyclic loading, the curve shows a constant increase in displacement. However, the impression that the displacement increases over-proportionately over the entire fatigue life is deceptive. The reason for the rising rate of increase in displacement is the periodic increase in loading (*S*_max_). Only considering the course of *S*_min_, it is noticeable that the curve is actually composed of a linear (up to about 80%) and a nonlinear part (80–100% of the fatigue life). This combination becomes even more obvious by looking at the confinement curve, which behaves in a similar way.

As already described in Section 4.2.2, the parameter ω provides little information about the actual damage of the fracture zone in the post-peak regime. In the pre-peak regime, however, the stiffness loss should give somewhat more information about the actual damage of the fracture zone. In Figure 8c, the stiffness reduction is plotted against the fatigue life. Up to about 80% of the fatigue life, no reduction in stiffness occurs. In fact, even a slight increase can be seen. After 80%, the stiffness decreases by about 10%. These results are in accordance with [46]. Here, this late decrease in stiffness is explained by the damage of the material structure, which does not occur until about 80% of the maximum shear strength. The concrete therefore basically remains uncracked up to this point. Consequently, the final fracture zone is formed only shortly before the maximum strength is reached. 

### 4.4. Fatigue Behavior under Constant Amplitudes

#### 4.4.1. General

**Figure 9:**
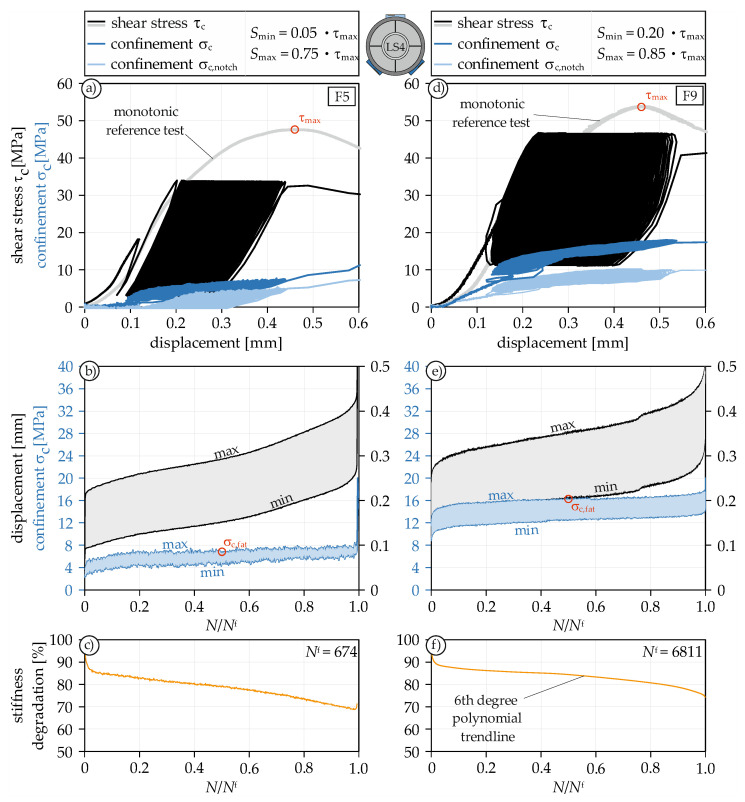
Stress-displacement curve under fatigue mode II loading (**a**,**d**), fatigue-displacement curve with corresponding confinement (**b**,**e**), and stiffness degradation (**c**,**f**) for F5 and F9.

To investigate the fatigue behavior of concrete under constant mode II loading, twelve fatigue tests were carried out and evaluated. A total of four different loading scenarios were performed, each featuring three repetitions. Since the specimens were cast in different concrete batches, prior to each loading scenario series, a monotonic reference test was conducted to determine the maximum shear strength τ_max_ (Figure 9a,d). Subsequently, the fatigue tests were performed, where the loading sinusoidally oscillated around a mean load. At the same time, the strain of the steel ring was measured with a maximum of 100 Hz, which was then used to determine the confinement. The stress-displacement curve with corresponding confinement of test F5 with *S*_min_ = 0.05 and *S*_max_ = 0.75 is shown in Figure 9a. The curve of test F9 with *S*_min_ = 0.20 and *S*_max_ = 0.85 is shown in Figure 9d.

In total, the tests F5 and F9 reached 674 and 6811 cycles, respectively. The maximum displacement of the last cycle was approximately at the same level for F5 as the displacement at maximum shear strength of the reference test. For the test with *S*_min_ = 0.20, the displacement exceeded the displacement at maximum shear load. As can be clearly seen in both Figures, there is a uniform loading and unloading behavior during the loading cycles in the confinement. The decrease in stiffness is relatively mild (Figure 9c,f). During the entire fatigue life of the concrete specimen, it dropped by only 25 to 30%. One reason for this small decrease certainly is the confinement, which compresses the shear surfaces and thus prevents a greater loss of stiffness. The fracture surfaces of the fatigue tests are visually indistinguishable from the fracture surfaces of the monotonic tests (Figure 5b). Besides the straight shear surface, no other fractures could be observed.

#### 4.4.2. Interaction of Fatigue Life and Confinement

Of particular interest in the present fatigue tests was the interaction of displacement and confinement. Figure 9b,e show the development of displacement and confinement as a function of fatigue life. Both curves show the typical S-shaped profile with a rapid increase in displacement in the first and third phase, which is already known from fatigue-creep curves of concrete specimens under compressive loading. Naturally, a higher *S*_max_ leads to larger maximum displacements per cycle and a higher *S*_min_ to larger minimum displacements. It is noteworthy that the confinement remains relatively constant in the second and longest phase. It can therefore be assumed that there is cyclic confinement loading with a constant maximum and minimum confinement during fatigue shear loading. Comparing the average maximum confinements with each other, F9 achieves a confinement almost three times as large as F5. This is because the dilation at 85% of the maximum shear strength is significantly more advanced than at 75%. Therefore, the confinement is considerably higher, too.

**Figure 10:**
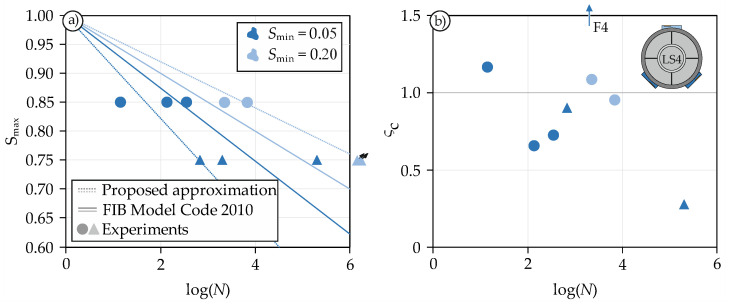
Wöhler curves (**a**) and confinement-lifetime-diagram (**b**) for the conducted experiments.

The ultimate fatigue life of all tests in relation to the upper load level *S*_max_ (Wöhler curve) is shown in Figure 10a. The graphs also depict the empirical approximation for pure compression according to FIB Model Code 2010 [59], which is permissible for log(*N*) ≤ 8 (Equations (5) and (6)).

(5) $$\log\left(N\right)=\frac{8}{\left(Y-1\right)}\cdot \left(S_{\max}-1\right)$$

(6) $$Y=\frac{0.45+1.8\cdot S_{\min}}{1+1.8\cdot S_{\min}-0.3\cdot S_{\min}{}^{2}}$$

Unfortunately, specimen F7 was pre-damaged. It failed after the first load cycle and has therefore been excluded from the evaluation shown in Figure 10. All tests of the LS4.4 series did not fail at all, even after more than 1.4 million cycles, and were therefore aborted prior to failure. A detailed summary of the results can be found in Table 3.

At first glance, the FIB Model Code 2010 approach gives an accurate estimation of the ultimate fatigue life. The mean value of each series is in the immediate vicinity of the approximation. However, a closer look reveals a considerable scatter within each series. To deepen the understanding of these scatterings, Figure 10b was compiled. This figure shows the ratio ς_c_ (Equation (7)) of the confinement under monotonic loading σ_c_ at the respective load level and the measured maximum confinement during the second phase of fatigue loading σ_c,fat_.

(7) $$\varsigma_{c}=\frac{\sigma_{c,\mathrm{fat}}}{\sigma_{c}}$$

To determine σ_c_, the monotonic reference tests were used. Naturally, the reference tests are also subject to certain scatter, yet an overall trend can still be identified with this method. Figure 10b reveals that some tests show significantly higher or lower confinement levels than expected. The reason probably is the inhomogeneity of the concrete, which causes the dilatation to occur at different rates, especially under fatigue loading, and thus the resulting confinement varies. Since the confinement is not controlled actively, but only induced passively by the steel ring, it is not possible to influence this loading during the test. However, through this natural, arbitrary variation of confinement, it was possible to establish a relationship between fatigue life and confinement. As Figure 10b indicates, the reason for the large scatter in the fatigue tests is the difference in confinement. This phenomenon is particularly evident in the tests F6 and F3. F6 exhibits only 28% of the monotonically measured confinement and thus achieves a lot more (201,740) cycles. In the case of F3, exactly the opposite effect can be observed. In this test, the confinement was about twice as high as in the other tests of this series, and the sample ultimately lasted only 14 cycles. The results of the LS4.4 series (*S*_min_ = 0.20/*S*_max_ = 0.75) are not plotted in Figure 10b, since no failure occurred. The dashed lines in Figure 10a indicate the approximation of the fatigue life under consideration of the confinement. These lines were generated assuming a confinement ratio of roughly ς_c_ = 1. The comparison to the predictions for concrete under compressive loading show that a variation in shear loading seems to have a significantly greater effect on the fatigue life than a variation in compressive loading. 

#### 4.4.3. Limitation of the Test Method

The shear strength under monotonic loading increases with increasing confinement [24,38]. This is due to the greater resistance of the fracture zone to displacements, if the shear surface is subjected to a compressive loading. This effect has two different consequences.

**Figure 11:**
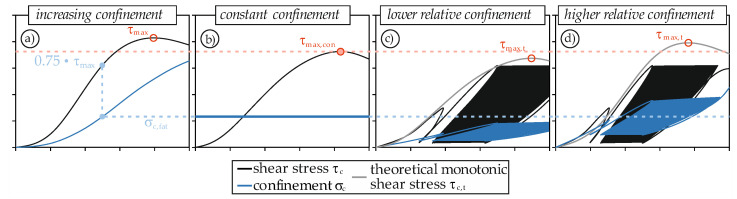
Schematic relationship between confinement and shear strength for increasing confinement (**a**), constant confinement (**b**), lower relative confinement (**c**), and higher relative confinement (**d**).

One consequence is the reduced reference shear strength τ_max_. Figure 11a schematically shows the stress-displacement curve with the corresponding confinement of a test with an outer steel ring. As already explained in Section 4.1.2, the confinement increases continuously. If the specimen is then loaded with a fatigue load (e.g., 0.75 · τ_max_), the maximum confinement remains relatively constant at σ_c,fat_ (Figure 9). If a monotonic test was then performed with this constant confinement σ_c,fat_, the maximum shear strength τ_max,con_ would be lower than τ_max_ (Figure 11b). The upper and lower loads *S*_max_ and *S*_min_ are therefore always related to the monotonic reference test. However, they do not provide any information about the actual utilization of the maximum shear strength of the specimen since there is a direct dependence between confinement and shear strength. 

The second consequence is underlined in Figure 11c,d. If the confinement during fatigue loading is lower/larger than assumed based on the reference test, the theoretical monotonic shear strength τ_max,t_ also decreases/increases. This additionally shifts the utilization of shear strength.

*S*_max_ and *S*_min_ would therefore have to be adjusted upwards for all tests to account for the first effect. Subsequently, all tests with ς_c_ > 1 (e.g., F3) would have to be adjusted downwards and all tests with ς_c_ < 1 (e.g., F6) upwards. Since the presented test setup does not allow active control of the confinement, it is not possible to experimentally determine the percentage of correction for the upper and lower loads of the individual tests. It is likely that instead of *S*_max_ = 0.75, it could rather be 0.85 to 0.90. Nevertheless, even without knowledge of the exact loading level, the results give a deep insight into the material behavior of high-strength concrete under mode II fatigue loading and allow for a qualitative evaluation of the influence of concurrent compressive loading and damage progression.

## 5. Conclusions

In this paper, a test setup that can be used to investigate the material behavior of concrete under compressive shear loading was presented. A comprehensive test program consisting of monotonic, cyclic, and fatigue tests was carried out and evaluated. The following results and conclusions can be drawn:

- The presented PTST setup is applicable for introducing steady compressive shear loading in a very straightforward manner without causing secondary cracks.
- With the help of the steel ring strain measurements, it is possible to determine the simultaneously acting compressive stress and thus correlate it with the shear strength. In addition, this allows to determine the nonlinear stress distribution along the specimen height.
- Size effect, which describes the influence of the specimen height on shear strength, could be systematically investigated and quantified. A statement about the shear strength of concrete should always be related to the specimen size or conducted using a specimen geometry that is not influenced by size effect.
- Low-cycle shear tests can be used for a systematic investigation of the loading and unloading behavior, even if the parameter ω is not suitable for defining the actual damage under compressive shear loading, due to the confinement, which compresses the shear surfaces even after the maximum shear strength is exceeded.
- By means of pre-peak cyclic tests, it could be shown that stiffness degradation is initiated at about 80% of the fatigue life, indicating that the development of the fracture zone starts rather late.
- The presented test setup provides the possibility to apply a controlled fatigue shear loading with simultaneously constant, measurable fatigue compressive loading.
- The results of the fatigue tests indicate that the variation of the upper/lower load in shear fatigue tests has a greater influence on the fatigue life than the variation of the upper/lower load in compression fatigue tests.
- The scattering of the results can be attributed to the confinement, which was not controllable and thus varied in each test.

The purpose of the presented investigations was to perform fatigue shear tests on high-strength concrete specimens as simply as possible. Motivation is the validation of a promising hypothesis [13] that represents the fatigue behavior on a thermodynamic basis, can be implemented in numerical models [60], and could serve as one element in complex, all-encompassing mechanical models such as [61,62]. The investigations showed that the clear influence of the confinement on the material behavior under monotonic loading is also of decisive importance in fatigue tests. Therefore, a final statement about the correctness of the aforementioned hypothesis is not yet possible. In future studies, this interaction should be systematically investigated in a targeted manner. For this purpose, a further development of the test setup would be advisable, allowing for control of the applied confinement. Alternatively, notched thin-walled tubes can be used, which allow independent control of axial and shear loading [37]. Furthermore, the difference between constant and cyclic compressive loading during shear fatigue loading should be analyzed. Finally, questions concerning damage degradation evaluation and the influence of sequence effects on the fatigue life under shear loading should be addressed.

## Figures and Tables

**Table 1:** Description of the loading scenarios.

Loading Scenario	Description	Purpose	Figure
LS1	Monotonic loading	Studying the monotonic behavior and identifying the shear strength	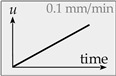
LS2	Cyclic loading	Providing detailed information on unloading and reloading in the post-peak regime	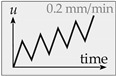
LS3	Cyclic step-wise increased loading	Providing detailed information on unloading and reloading in the pre-peak regime	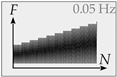
LS4	Fatigue loading with constant amplitudes	Characterizing the concrete fatigue behavior under constant amplitudes	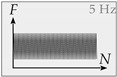

**Table 2:** Test matrix (monotonic reference tests in brackets).

Loading Scenario	LS1	LS2	LS3	LS4.1	LS4.2	LS4.3	LS4.4
***S*_max_**	-	-	0.5 + *n* ⋅ 0.05	0.85	0.75	0.85	0.75
***S*_min_**	-	-	0.05	0.05	0.05	0.20	0.20
**Repetitions**	8 + 17	2	2 (1)	3 (1)	3 (1)	3 (1)	3 (1)

**Table 3:** Summary of all conducted tests of the series LS4.

Test	Loading Scenario	*S*_max_	*S*_min_	Number of Cycles *N*^f^	Confinement Ratio ς_c_
F1	LS4.1	0.85	0.05	350	0.73
F2	LS4.1	0.85	0.05	135	0.66
F3	LS4.1	0.85	0.05	14	1.17
F4	LS4.2	0.75	0.05	1998	2.78
F5	LS4.2	0.75	0.05	674	0.9
F6	LS4.2	0.75	0.05	201,740	0.28
F7	LS4.3	0.85	0.20	1	-
F8	LS4.3	0.85	0.20	2232	1.09
F9	LS4.3	0.85	0.20	6811	0.96
F10	LS4.4	0.75	0.20	1,669,000	-
F11	LS4.4	0.75	0.20	1,469,000	-
F12	LS4.4	0.75	0.20	1,739,359	-

## Data Availability

The data presented in this study are available on request from the corresponding author.
